# Draft genome sequences of six *Pseudomonas* spp. and one *Rheinheimera* sp. isolated from oil sands process-affected water from Alberta, Canada

**DOI:** 10.1128/MRA.00589-23

**Published:** 2023-11-15

**Authors:** Kira L. Goff, Jeff Gauthier, Steven M. Shideler, Tyson Bookout, Irena Kukavica-Ibrulj, Shawn Lewenza, Roger C. Levesque

**Affiliations:** 1 Department of Microbiology, Immunology and Infectious Diseases, University of Calgary, Alberta, Canada; 2 Faculty of Science and Technology, Athabasca University, Athabasca, Alberta, Canada; 3 Institute of Integrative and Systems Biology, Laval University, Quebec, Canada; University of Delaware College of Engineering, Newark, Delaware, USA

**Keywords:** oil sands, bioremediation, bitumen, *Pseudomonas*, *Rheinheimera*

## Abstract

We report the draft genomes of seven bacterial strains (six *Pseudomonas* spp. and one *Rheinheimera* sp.) isolated from environmental water samples from oil sands tailings ponds that have accumulated a wide variety of organic compounds, salts and metals.

## ANNOUNCEMENT

Bitumen surface mining in Canada’s Athabasca oil sands regions results in the production of large volumes of oil sands process water (OSPW), containing a complex mix of organic and inorganic compounds such as polycyclic aromatic hydrocarbons, naphthenic acid fraction components, and BTEX (benzene, toluene, ethyl benzene, and xylenes) (1). Understanding microbial survival in OSPW is the first step in developing bioremediation strategies. Here, we present the draft genome sequences of seven bacteria (six *Pseudomonas* spp. and one *Rheinheimera* sp.) isolated from OSPW samples obtained from an operator in the Athabasca oil sands region. Samples of OSPW were spread plated on Pseudomonas Isolation Agar (BD Difco) and then incubated at room temperature. Single colonies were isolated and purified by serial streaking.

For each strain, pure culture isolates were obtained by plating on Brain-Heart Infusion (BHI) agar (BD Difco) at room temperature (~25°C) for 48 h. A pure colony was transferred in 2 mL of BHI broth until OD_600_ reached 1.0. Cells were collected by centrifugation at 10,000 × *g* for 20 min at 4°C, and DNA was extracted from pellets using the DNeasy Blood & Tissue kit (QIAGEN) with the manufacturer’s recommended protocol for Gram-negative bacteria. DNA was quantified with the Qubit dsDNA BR kit (Thermo Fisher) and assessed for purity (OD_260_/OD_280_ > 1.8) with a NanoDrop 2000 spectrophotometer (Thermo Fisher). Then, 1 µg genomic DNA was sheared in 400–700 bp fragments with the G-tube protocol (Covaris), after which DNA was sequenced in paired ends (2 × 300 bp) on a MiSeq apparatus (Illumina) using the TruSeq 3 PE chemistry. Read quality was checked with FastQC version 0.11.8 (2), and adapter sequences were trimmed with TRIMMOMATIC version 0.39–2 (3). Trimmed Illumina reads were assembled with the A5 pipeline version 20150522 with default parameters (4). Assembly and coverage metrics (e.g., number of reads, estimated genome size, GC%, etc.) have been included in Table 1.

**TABLE 1:** Main assembly metrics and number of features for each assembled genome^*a*^

Isolate ID	Organism	dDDH d4 (CI, %)	Reads sequenced	Reads passing QC	Raw coverage (×)	Genome size (Mbp)	No. scaffolds	Scaffold N50 (bp)	Completeness (%)	GC content (%)	# CDS	# rRNAs	# tRNAs
2805	Pseudomonas extremaustralis 1906	92.5 (90.4–94.1)	1,070,891	736,36,	29	6,30	121	114,648	100	57	5,854	51	107
2808	Pseudomonas proteolytica 1912-L	89.4 (87.0–91.4)	1,670,064	1,059,741	47	5,96	86	168,052	99	58	5,318	57	105
2809	Pseudomonas sp. 1912-s	48.7 (46.1–51.3)	1,845,977	901,083	44	7,27	83	186,061	100	55	6,452	47	98
2810	Pseudomonas paracarnis 1923	94.0 (92.2–95.4)	1,842,031	1,046,203	47	6,26	47	342,875	100	54	5,734	54	108
2811	Rheinheimera sp. 1928-s	54.4 (51.7–57.1)	794,464	734,898	36	4,64	24	599,252	100	45	4,152	55	124
2812	Pseudomonas sp. 1928-m	42.2 (39.7–44.8)	670,112	581,556	33	4,40	17	490,049	100	59	4,056	49	103
2813	Pseudomonas veronii 1929	89.0 (86.6–91.0)	1,017,500	699,134	35	6,62	114	96,808	100	57	6,073	58	103

^*a*^ dDDH d4: proportion of sequence identity matched between draft genome and the most similar reference genome. Raw coverage: average number of reads covering each nucleotide position in the assembly. Completeness: an estimate calculated by CheckM, based on the presence of core genes, and the consistency of nucleotide usage statistics across scaffolds. CDS: coding DNA sequences. rRNAs: ribosomal RNA genes. tRNAs: transfer RNA genes.

Annotation was done with version 6.4 of the NCBI Prokaryotic Genome Annotation Pipeline (5). Taxonomy was assigned to draft genomes using the Type Strain Genome Server via whole genome *in silico* digital DNA-DNA hybridization (dDDH) (6). These include three potential new species (dDDH < 70%).

Additional genomic analysis of these strains will facilitate a greater understanding of the molecular mechanisms of resistance to the toxicants present in OSPW.

## Data Availability

These draft genome sequences have been deposited in DDBJ/ENA/GenBank and raw reads in the Sequence Read Archive (SRA) under NCBI BioProject PRJNA941309. Accession numbers are SRR25168148, SRR25168145, SRR25168144, SRR25168143, SRR25168142, SRR25168141, and SRR25168140 (SRA) and JARIXU000000000, JARIXS000000000, JARIXR000000000, JARIXQ000000000, JARIXP000000000, JARIXO000000000, and JARIXN000000000 (NCBI Genome).

## References

[1] Li C, Fu L, Stafford J, Belosevic M, Gamal El-Din M. 2017. The toxicity of oil sands process-affected water (OSPW): a critical review. Sci Total Environ 601–602:1785–1802. doi:10.1016/j.scitotenv.2017.06.024 28618666

[2] Andrews S. 2010. FastQC: a quality control tool for high throughput sequence data. Available from: https://www.bioinformatics.babraham.ac.uk/projects/fastqc

[3] Bolger AM, Lohse M, Usadel B. 2014. Trimmomatic: a flexible trimmer for Illumina sequence data. Bioinformatics 30:2114–2120. doi:10.1093/bioinformatics/btu170 24695404 PMC4103590

[4] Tritt A, Eisen JA, Facciotti MT, Darling AE. 2012. An integrated pipeline for de novo assembly of microbial genomes. PLoS One 7:e42304. doi:10.1371/journal.pone.0042304 23028432 PMC3441570

[5] Tatusova T, DiCuccio M, Badretdin A, Chetvernin V, Nawrocki EP, Zaslavsky L, Lomsadze A, Pruitt KD, Borodovsky M, Ostell J. 2016. NCBI prokaryotic genome annotation pipeline. Nucleic Acids Res 44:6614–6624. doi:10.1093/nar/gkw569 27342282 PMC5001611

[6] Meier-Kolthoff JP, Göker M. 2019. TYGS is an automated high-throughput platform for state-of-the-art genome-based taxonomy. Nat Commun 10:2182. doi:10.1038/s41467-019-10210-3 31097708 PMC6522516

